# Effect of Strength-Based Resilience on Patient's Length of Stay at the Renfrew Center for Eating Disorders

**DOI:** 10.1089/whr.2022.0044

**Published:** 2022-09-29

**Authors:** Kaitlin Sanzone, Daniel Short, John Gaughan, Lori Feldman-Winter

**Affiliations:** ^1^ Department of Education, Cooper Medical School of Rowan University, Camden, New Jersey, USA.; ^2^ Department of Medicine, Cooper University Hospital, Camden, New Jersey, USA.; ^3^ Division of Adolescent Medicine, Department of Pediatrics, Children's Regional Hospital, Cooper University Health Care, Cooper Medical School of Rowan University, Camden, New Jersey, USA.

**Keywords:** eating disorder, anorexia, bulimia, mindfulness, resilience, length of stay

## Abstract

**Objective:**

Throughout past decades, physicians have sought to understand factors that contribute to severity of an eating disorder (ED). There is a potential relationship between patients' resilience and the recovery course of their disorder. The objective of this study is to examine the correlation between resilience, measured by indicators of mindfulness and restraint, and length of stay (LOS) at Renfrew Center for Eating Disorders.

**Materials and Methods:**

Data were obtained from Renfrew's database. Secondary analysis was conducted from this database. The database included women aged 13–75 years admitted to Renfrew. The database excluded males and individuals of ages <13 or >75 years old. Resilience was analyzed through Southampton Mindfulness Questionnaire (SMQ) and ED restraint. The dependent variable, LOS at Renfrew, was analyzed by multivariable linear regression, and multivariable logistic regression for LOS >45 days.

**Results:**

A sample of 2901 subjects was analyzed. There were significant associations between increased mindfulness scores and decreased restraint scores and a decreased LOS at Renfrew. For every 13-point increase in SMQ, the LOS was associated with a decrease by ∼1 day, and for every 1-point decrease in the restraint score (increased restraint), the LOS was associated with a decrease by ∼1 day. Higher restraint scores were independently associated with an increase in likelihood of LOS >45 days by 22.8%.

**Conclusions:**

The Renfrew data support the relationship between a patient's resilience and LOS. This information holds promise for future treatment approaches to improve strength-based modalities in patients suffering from EDs.

## Introduction

Throughout past decades, psychiatrists and researchers have sought to identify factors that contribute to the severity of an eating disorder (ED). The etiology of EDs including anorexia nervosa, bulimia nervosa, binge ED, and atypical EDs that affect many young women, and even men, is complex and varied. Known risk factors for developing an ED include a family history of an ED, mental illnesses such as anxiety, depression, or obsessive-compulsive disorders, prior trauma including sexual, physical, and emotional abuse, and characteristics of low self-esteem, perfectionism, impulsive behavior, and troubled relationships.^1^

Although the amount of known risk factors contributing to the development of EDs is increasing, evidence for determining the severity of the ED remains inconclusive.^2^

The literature supports an association between negative self-image, feelings of being rejected by others, and resilience among patients with anorexia nervosa.^3^ There is also evidence from a clinical population of adolescents that body size dissatisfaction is a predictor of low resilience.^4^ In a qualitative study of adults recovering from EDs, authors suggest a multilevel resilience framework as a potential strategy for treatment and recovery.^5^ The goal of our study was to further understand the relationship between mindfulness, a strength-based modality, and restraint and a patient's recovery.

Given that resilience can be improved by strength-based counseling strategies, we sought to determine whether indicators of low resilience were associated with the length of stay (LOS) among inpatients with EDs, with LOS serving as a further association to severity of disease based on how long it takes a patient to recover sufficiently for treatment center discharge. Our hypothesis was that low levels of resilience, measured by surveys of mindfulness and restraint, are independently associated with increased LOS among hospitalized patients with EDs. By researching these associations, potential novel targets for early intervention in patients at risk for, or currently suffering from, an ED can be implemented.

## Materials and Methods

### Sample and setting

We used an observational cohort study design with data obtained from The Renfrew Center for Eating Disorders' database. Patients included in the database were those who were admitted to Renfrew's inpatient facility, female gender, and ages 13–75 years. Those excluded from the database were those of ages <13 years or >75 years and male gender, as Renfrew only admits patients that are cis-gendered females. These age groups were excluded to focus analyses on the primary age range and demographics of patients admitted to Renfrew inpatient facilities, located in many areas across the east coast and midwest regions of the United States. The final sample included subjects based on completion of both resilience scales upon admission to Renfrew.

The sample size available through the Renfrew database of at least 3000 inpatients provided >99% power to detect a correlation coefficient of 0.1 or greater based on a *p*-value of 0.01 (two-tailed) and provided adequate power for higher order relationships to be explored.

### Measures

Demographic variables including race, employment, education, and trauma were self-reported and the type of ED was diagnosed through the Renfrew clinicians. The independent variables available to assess resilience included mindfulness, one aspect of resilience, using the Southampton Mindfulness Questionnaire (SMQ) as well as the Eating Disorder Restraint Questionnaire (EDERESTRNT). These self-reported surveys were administered upon admission to Renfrew.

The SMQ assesses the relationship one establishes with distressing thoughts and images. One study in 2008 explored the reliability and validity of the SMQ for research purposes. The data supported the use of the SMQ in both clinical practice and in research to assess mindful responding to distressing thoughts. Therefore, this study uses SMQ to serve as a link between mindfulness and resilience.^6^ The survey includes 16 items scored on a 7-point Likert scale, worded “strongly disagree” (0) to “strongly agree” (6), yielding a total range of 0–96.

For SMQ, the higher their score, the more mindful the patient. In the general population, the mean SMQ value was found to be 49.3 (standard deviation [SD] = 15.20).^7^ The current literature analyzing the reliability and validity of the SMQ states that the questionnaire had strong internal consistency and adequate concurrent validity.^6^

EDERESTRNT is an eating questionnaire that assesses different aspects of restraint and is scored 0 to 6 with a higher score correlating with less restraint. The restraint norms based on data from a community-based sample of 241 women showed the mean restraint to be 1.251 (SD = 1.323).^8^

The outcome variable, LOS at Renfrew facility, was obtained from Renfrew's database. Patient's LOS was recorded by Renfrew staff. This outcome variable was intended to serve as a marker of severity of disease, with longer LOS correlating to more severe ED.

### Statistical analysis

Mindfulness and restraint scores as predictors of LOS were analyzed as continuous variables. Regression analysis was completed independently for both restraint and SMQ. In the first analysis, we used LOS as a continuous measure and conducted a multivariable linear regression controlling for additional independent variables, including race/ethnicity, employment, education, and type of ED. Second, we conducted a univariate logistic regression analysis with bivariate outcome that was selected based on mean LOS plus 1 SD. The LOS was dichotomized into short versus long (>45 days) inpatient stays for subjects with EDs, as this was 1 SD above Renfrew's mean LOS.

Furthermore, we conducted a multivariable logistic regression controlling for the demographic characteristics already noted. These analyses provided us with unadjusted and adjusted odds ratios presented with 95% confidence intervals (CIs).

#### Institutional Review Board

The study was authorized by the Renfrew Center for Eating Disorders and reviewed and approved by the Cooper University Hospital's institutional review board with waiver of consent.

## Results

**FIG. 1.**
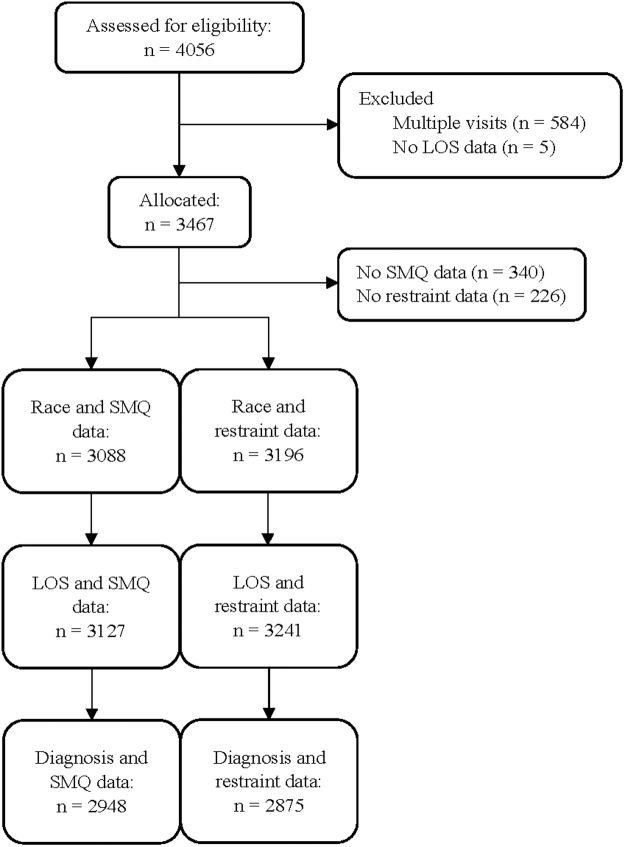
Eligibility and allocation of patient data used for the study. The analysis was divided based on whether SMQ and/or restraint data were available per patient. SMQ, Southampton Mindfulness Questionnaire.

A sample size of 3,467 individuals was allocated to the analysis based on inclusion and exclusion criteria, yielding a final sample for analysis of 2901 patients. Figure 1 illustrates the selection of subjects using guidelines developed by the Strengthening the Reporting of Observational Studies in Epidemiology.^9^

Table 1 highlights the demographic characteristics of the patients analyzed. In our sample and throughout the literature, Caucasian race was the most common among those hospitalized for EDs when compared with other races.^10^ Furthermore, this sample of patients with EDs showed higher rates of unemployment, yet the rates of student status and working full time were nearly as high. Lastly, over half of our sample showed education level at a college degree or higher. Bulimia nervosa and anorexia nervosa were the most common types of EDs affecting hospitalized patients at Renfrew.

**Table 1.** Demographics of the Patient Population at Renfrew Center for Eating Disorders

Baseline characteristics	N	%	SMQ Mean	SMQ SD	EDERESTRNT Mean	EDERESTRNT SD	LOS Mean	LOS SD
Race/Ethnicity	3283	—	32.5	16.9	3.41	1.98	30.8	15.4
Caucasian	2737	83.4	30.9	16.6	3.58	1.93	31.3	14.4
African American	62	1.9	32.9	19.5	3.60	2.09	29.9	12.2
Asian	71	2.2	32.1	14.0	3.30	1.92	30.4	13.8
Hispanic	200	6.1	32.6	17.4	3.38	1.97	31.8	17.7
Native American	19	0.58	33.6	15.6	3.24	2.06	29.1	16.9
Other	194	5.91	33.0	17.9	3.38	1.92	32.4	16.5
Employment	3278	—	31.5	17.0	3.54	1.90	31.0	14.4
Unemployed	1043	31.8	32.5	16.9	3.35	2.02	32.2	16.7
Part time	466	14.2	31.3	15.7	3.71	1.95	30.1	14.1
Full time	804	24.5	29.8	16.7	3.67	1.81	30.4	13.2
Student	850	25.9	30.8	16.4	3.67	1.81	32.4	14.0
Other	115	3.5	33.1	19.2	3.31	1.91	30.2	13.9
Education	3303	—	31.1	16.8	3.56	1.95	31.2	14.6
<High school	841	25.5	32.9	16.2	3.47	1.94	34.0	15.6
High school	313	9.48	31.7	16.7	3.47	2.04	30.6	14.5
College	1588	48.1	30.7	16.7	3.57	1.91	30.6	14.4
>College	346	10.5	29.5	16.8	3.73	1.88	30.3	12.8
Other	215	6.50	30.9	17.8	3.57	1.98	30.7	15.7
ED	3066	—	32.6	16.4	2.81	1.68	31.2	15.0
Anorexia nervosa	736	24.0	32.2	16.8	3.46	1.93	37.1	17.0
Anorexia purging	588	19.2	31.0	16.0	4.06	1.71	32.9	16.2
Bulimia nervosa	839	27.4	29.8	17.0	3.57	1.87	28.7	11.3
Binge ED	172	5.6	29.6	16.5	1.33	1.46	26.0	11.3
ARFID	54	1.76	43.5	16.3	0.52	1.07	32.5	19.6
Other	677	22.1	29.3	16.1	3.94	1.87	30.0	12.8

Characteristics of the patient study sample at Renfrew allocated by baseline mindfulness and restraint scores, and background information that includes race/ethnicity, employment, education level, and ED diagnosis.

ARFID, avoidant-restrictive food intake disorder; ED, eating disorder; LOS, length of stay; EDERESTRNT, Eating Disorder Restraint Questionnaire; SD, standard deviation; SMQ, Southampton Mindfulness Questionnaire.

The mean mindfulness score (SMQ) was 31.25 (SD = 16.66) and the mean resilience score (EDERESTRNT) was 3.55 (SD = 1.94). Education level and the type of ED were statistically different regarding LOS at Renfrew, using chi-squared analysis. However, the race/ethnicity of patients was not statistically different regarding LOS. Associations between mindfulness, restraint, additional demographics, and LOS were tested using multiple linear regression (Table 2).

**Table 2.** Multiple Linear Regression Analysis for Associations with Length of Stay Using Backward Selection Accounting for All Statistically Significant Confounding Variables

	Estimate	95% CI	***p***
SMQ	−0.0793	−0.113 to −0.046	<0.0001
EDRESTRNT	0.850	0.544–1.16	<0.0001
>College	−3.45	−5.36 to −1.53	0.001
College	−2.90	−4.73 to −2.16	<0.0001
High school	−2.10	−4.14 to −0.0622	0.043
Other education	−1.51	−3.78–0.768	0.194
Anorexia, purging type	−4.52	−6.16 to −2.88	<0.0001
ARFID	−2.42	−6.66–1.81	0.262
Binge ED	−9.01	−11.6 to −6.46	<0.0001
Bulimia nervosa	−7.77	−9.25 to −6.28	<0.0001
Other ED	−7.04	−8.59 to −5.48	<0.0001

All other variables that were not statistically significant were not included in this analysis.

CI, confidence intervals; EDERESTRNT, Eating Disorder Restraint Questionnaire.

Increased mindfulness scores and decreased restraint scores were significantly associated with a decreased LOS; SMQ estimate −0.08 (95% CI −0.11 to −0.05; *p* < 0.0001), and EDERESTRNT estimate 0.85 (95% CI 0.55–1.16l; *p* < 0.0001). For every 13-point increase in the mindfulness questionnaire, the LOS was associated with a decrease by ∼1 day [LOS = −0.08 (SMQ) +33.7; *r*^2^ = 0.01]. Similarly, another regression plot for restraint scores and LOS was performed, indicating the association between restraint scores and LOS at Renfrew. For every 1-point decrease in the restraint questionnaire, the LOS was associated with a decrease by ∼1 day [LOS = −1.11 (EDERESTRNT) +27.5; *r*^2^ = 0.02]. There were also significant associations between education, and specific types of ED, and LOS.

We also analyzed the associations between mindfulness and restraint and other independent variables. There were no statistically significant associations between race/ethnicities of patients for both the mindfulness and restraint score (data not shown). Furthermore, we found significant associations in mindfulness but not restraint scores between those with education less than high school versus those with college education and beyond.

Patients in our sample had a mean LOS of 31.62 days (SD = 14.82) with a median of 45 days. Using univariable and multivariable logistic regression (Table 3), there was a slightly lower unadjusted odds ratio of 0.988 (95% CI 0.981–0.994; *p* = 0.0003) and an adjusted odds ratio of 0.978 (95% CI 0.970–0.985; *p* < 0.0001) for higher LOS (>45 days) associated with higher levels of mindfulness. Similarly, there was an increased unadjusted odds ratio of 1.90 (95% CI 1.123–1.268; *p* < 0.0001) and an adjusted odds of 1.228 (95% CI 1.145–1.317; *p* < 0.0001) for higher LOS associated with higher restraint scores (lower levels of restraint).

**Table 3.** Association Between Mindfulness, Restraint, and Other Variables and Outcome of Length of Stay >45 Days Versus 45 Days or Less

	***n***	Odds ratio	95% CI	***p***	Adjusted odds ratio^a^	95% CI	***p***
EDRESTRNT		1.19	1.12–1.27	<0.0001	1.228	1.15–1.32	<0.0001
Race/ethnicity	2867						
White, non-Hispanic	2395	Ref	—	—	Ref	—	—
African American	58	0.483	0.174–1.34	0.162	1.23	1.15–1.32	<0.0001
Asian	62	1.02	0.501–2.06	0.964	0.651	0.229–1.85	0.421
Hispanic	173	1.10	0.717–1.67	0.679	1.05	0.462–2.37	0.913
Native American	16	1.31	0.381–4.53	0.667	1.06	0.224–4.97	0.945
Other	163	1.33	0.892–1.99	0.161	1.49	0.952–2.32	0.081
Education	2877						
<High school	692	Ref	—	—	Ref	—	—
>College	308	0.573	0.392–0.838	0.00410	0.616	0.383–0.990	0.045
College	1415	0.568	0.448–0.720	<0.0001	0.655	0.492–0.873	0.004
High school	267	0.800	0.559–1.146	0.2241	0.947	0.623–1.44	0.797
Other	195	0.476	0.291–0.780	0.0032	0.601	0.345–1.05	0.072
ED diagnosis	2659						
Anorexia nervosa, nonpurging	551	Ref	—	—	Ref	—	—
Anorexia, purging	504	0.496	0.373–0.660	<0.0001	0.489	0.357–0.670	<0.0001
ARFID	43	0.762	0.385–1.51	0.4351	1.54	0.716–3.308	0.269
Binge ED	166	0.108	0.047–0.247	<0.0001	0.166	0.066–0.419	<0.0001
Bulimia nervosa	786	0.201	0.145–0.278	<0.0001	0.219	0.154–0.311	<0.0001
Other	609	0.330	0.246–0.449	<0.0001	0.324	0.234–0.449	<0.0001

One SD above Renfrew's mean LOS for EDs is 45 days. In a separate multivariable analysis adjusted for covariates, the odds ratio for SMQ was 0.988 (95% CI 0.981–0.994; *p* = 0.0003), with an adjusted odds ratio of 0.978 (95% CI 0.970–0.985; *p* < 0.0001).

^a^ Variables included in the model were EDERESTRNT, race, employment, education, and diagnosis.

EDERESTRNT, Eating Disorder Restraint Questionnaire; Ref, referent.

Furthermore, African American patients had lower odds of LOS >45 days (0.483) compared with Caucasian patients (referent). When using less than high school education as a referent, all other education levels lowered the odds of higher LOS. Finally, when using anorexia nervosa as a referent, almost all other diagnoses were associated with a lower odds of LOS >45 days. The only exceptions included avoidant-restrictive food intake disorder that was not associated with prolonged LOS and binge-eating disorder that was associated with a slightly higher odds of increased LOS when compared with anorexia nervosa.

## Discussion

Among this sample of patients identified from the Renfrew Center's database, mindfulness and restraint, two aspects of resilience, are statistically associated with LOS. The mean mindfulness scores for our sample population were lower by ∼18 points than the general population, and the restraint scores were 2.3 points (over two and a half times) higher, indicating low levels of restraint upon admission to Renfrew.^7,8^ This demonstrates overall more severe behaviors and potentially lower resilience among Renfrew patients at the onset of their admission.

We expect that any interventions received during the patient's stay at Renfrew accounted for the baseline mindfulness and restraint scores. Furthermore, as these scores were obtained at the time of admission, these findings may indicate which patients at the time of admission warrant more focus on strength-based counseling approaches.

The findings in this study further our understanding of the association between mindfulness, resiliency, and ED recovery. Although the effect size for the association between mindfulness and LOS was smaller than what we expected to find, this finding may reflect interventions throughout the hospital stay aimed at improving mindfulness. Restraint at admission, however, was more robustly associated with LOS, and may indicate less change throughout the hospital stay.

The current literature, including systematic reviews and meta-analyses, supports these findings in other medical settings.^11^ For example, one study suggests that body dissatisfaction, binge eating, and emotional/exertional eating specifically had the strongest relationship between mindfulness and ED psychopathology.^4^ Other authors note that employment has been linked to improvements in mental health status and can even facilitate recovery from mental illness due to its ability to provide self-sufficiency, give individuals a purpose, and increase self-esteem.^12^

Therefore, this new relationship between mindfulness and restraint and LOS is a potentially important contribution to the current ED research and may have implications for avoidance of hospitalization or reduction in treatment center stay.^13^ Our findings indicate that although mindfulness and restraint both significantly predict patients' LOS, restraint is a more impactful predictor than mindfulness as presented in the adjusted odds ratios from the multivariable analyses. Having a higher restraint score, less restraint, increased the likelihood of LOS >45 days by 22.8%.

In addition, the findings that resilience is significantly related to LOS at inpatient ED facilities suggest that mindfulness and restraint, which can be improved by strength-based counseling strategies, may be a useful modality to incorporate in treatment plans of patient with an ED, specifically those of binge ED and bulimia nervosa.^14^ In the United States, these are also the most common subtypes of EDs, reflecting a representative sample at Renfrew.^8^ Current and previous clinical trials indicate that incorporating mindfulness and resiliency techniques may provide a suitable avenue of care in patients with EDs.^5,15–17^ The authors note that use of these techniques can improve quality of life while optimizing public resources in treatment of EDs.

There were several limitations to this study. The results of this study do not prove causality such that an increase in mindfulness or general resilience results in lesser severity EDs or decreased LOS, but rather we are operating under the assumption that the “Bradford Hill's criteria” were met, despite its limitations.^18^ The specific criteria met by this observational study include temporality, strength of the relationship, biological plausibility, alternative explanations, consistent with other knowledge, and specificity of the association. The criteria that require future research include replication and cessation of exposure, which may be achieved by repeated measures using SMQ and restraint scores throughout the inpatient treatment program.

Mindfulness and restraint scores were assessed through self-report at the time of admission, which may have led to inaccurate reporting depending on subjects' understanding of their actions and willingness to share this information. In addition, ED diagnoses may have not been consistent given that there was a switch from clinically incorporating DSM-IV criteria to DSM-5 criteria while data was being collected. Although diagnoses were put into groups to ameliorate this effect, it is possible that the diagnoses were not exactly equivalent. However, it is important to note that after this transition, all ED diagnoses were defined by clinicians at Renfrew using the DSM-5 criteria.

Despite these limitations, the present findings provide a systematic and statistically rigorous contribution to the understanding of mindfulness and resiliency with ED inpatient outcomes. Future research should study the causative relationships by applying a range of techniques to improve mindfulness and restraint to determine whether this can decrease ED LOS. Lastly, measures of mindfulness can be tested in the ambulatory setting to determine severity and likelihood of remission for patients with ED.^19^ If these associations hold true, mindfulness-based modalities can be incorporated into ED treatment plans as a method to avoid hospitalization.

## Conclusions

Our findings using data from the Renfrew Center for ED support the relationship between patient's resilience involving mindfulness and restraint and their length of hospitalization. This information holds promise for future treatment approaches involving strength-based modalities for inpatients with ED. This route of psychotherapy holds potential to be used alongside other modalities of treatment to improve patient outcomes more optimally for EDs.

## Abbreviations Used

ARFID: avoidant-restrictive food intake disorder
CI: confidence intervals
ED: eating disorder
EDERESTRNT: Eating Disorder Restraint Questionnaire
LOS: length of stay
Ref: referent
SD: standard deviation
SMQ: Southampton Mindfulness Questionnaire
SD: standard deviation
